# Impact of Umbilical Cord Milking on Hematological Parameters in Preterm Neonates With Placental Insufficiency

**DOI:** 10.3389/fped.2021.827219

**Published:** 2022-03-04

**Authors:** Mohammed Nagy, Nehad Nasef, Ahmed Gibreel, Mohamed Sarhan, Hoda Aldomiaty, Mohammed Darwish, Islam Nour

**Affiliations:** ^1^Neonatal Intensive Care Unit, Mansoura University Children's Hospital, Mansoura, Egypt; ^2^Departement of Pediatrics, Faculty of Medicine, University of Mansoura, Mansoura, Egypt; ^3^Department of Obstetrics and Gynecology, Faculty of Medicine, University of Mansoura, Mansoura, Egypt; ^4^Hematology Unit, Mansoura University Children's Hospital, Mansoura, Egypt; ^5^Departement of Clinical Pathology, Faculty of Medicine, University of Mansoura, Mansoura, Egypt

**Keywords:** preterm neonate, placental insufficiency, stem cell, umbilical cord, milking

## Abstract

**Background:**

Data is still lacking about the expediency of umbilical cord milking (UCM) in preterm neonates born to mothers with placental insufficiency (PI).

**Objective:**

To study the effect of UCM in preterm neonates who had ante-natal evidence of placental insufficiency on peripheral blood cluster of differentiation 34 (CD34) percentage, hematological indices, and clinical outcomes.

**Methods:**

Preterm neonates, <34 weeks' gestation, born to mothers with evidence of placental insufficiency that underwent UCM (PI+UCM group) were compared with historical controls whose umbilical stumps were immediately clamped [PI+ICC (immediate cord clamping) group] in a case-control study. Peripheral blood CD34 percentage as a measure of hematopoietic stem cell transfusion was the primary outcome. Early and late-onset anemia; polycythemia; frequency of packed red blood cells (PRBCs) transfusion during NICU stay; peak total serum bilirubin (TSB); incidence of phototherapy, admission rectal temperature; first 24 h hypothermia and hypoglycemia; episodes of hypotension and need for volume expander boluses and inotropic support during the first 24 h of age; duration of oxygen therapy; bronchopulmonary dysplasia (BPD); severe intra-ventricular hemorrhage (IVH); necrotizing enterocolitis (NEC); culture-proven late-onset sepsis; length of hospital stay; and in-hospital mortality were secondary outcomes.

**Results:**

In preterm infants with placental insufficiency, umbilical cord milking was associated with greater peripheral blood CD34 percentage, hemoglobin levels initially and at postnatal age of 2 months, alongside significantly shorter duration of oxygen therapy compared with ICC group. Frequency of packed RBCs transfusion during hospital stay was comparable. Neonates in UCM group had a greater peak TSB level during admission with significantly higher need for phototherapy initiation compared with ICC. Logistic regression, adjusted for gestational age, revealed that UCM resulted in greater CD34 percentage, higher initial hemoglobin level, higher peak serum bilirubin, significant increase of phototherapy initiation, and higher hemoglobin level at 2 months.

**Conclusions:**

UCM in preterm neonates born to mothers with placental insufficiency was feasible and resulted in greater CD34 percentage, higher initial hemoglobin level, higher peak serum bilirubin, significant increase of phototherapy initiation, and higher hemoglobin level at 2 months.

## Introduction

Placental transfusion is a transfer of residual placental blood to a newborn after birth till the time of umbilical cord clamping and cutting. Three recent meta-analyses revealed that placental transfusion, compared with ICC, was associated with reduced incidence of mortality, IVH, and need for blood transfusions for infants born preterm (1–3). This additional blood volume may exert its beneficial effects by enhancing neonatal iron-rich cell volume (4), improving cardiovascular hemodynamics (5), and transferring progenitor stem cells that may replace damaged cells and ameliorate immunocompetence (6). Most of clinical practice guidelines endorsed deferred clamping the cord at birth for variable timing as a standard of care for stable preterm (7–9). UCM has been considered as an alternative method to deferred cord clamping (DCC) particularly if immediate resuscitation is deemed necessary or in situations with unfavorable feto-maternal circulation hemodynamics. Previous studies showed that UCM in preterm infants provides cardiovascular stability as evidenced by greater systemic blood flow, higher left diastolic function, and improved cerebral perfusion compared to ICC (10, 11). In contrast, an experimental study showed that preterm lambs that underwent UCM had large cerebral blood flow fluctuations and may be susceptible to IVH. However, none of fetal lambs received antenatal steroid (12).

Preterm neonates with IUGR are more liable to various perinatal complications compared to those adapted for gestational age including hypothermia, hypoglycemia, IVH, NEC, sepsis, hyperbilirubinemia, polycythemia, BPD, and mortality (13). In addition, they are at high risk to have long term neurodevelopmental disability and cognitive delay (14). In most of the previously published placental transfusion studies, infants with IUGR or antenatal evidence of placental insufficiency have been excluded for the assumed risks of delayed resuscitation and aggravating polycythemia. Wang et al. (15) performed a subgroup analysis of preterm infants with IUGR who were randomized within their original trial of DCC vs. ICC and concluded that DCC has no harmful effects. Furthermore, the results of our previously published randomized controlled trial denoted that DCC in infants with IUGR was associated with mounted stem cell transfusion and greater hemoglobin levels at 2 months of age without higher incidence of polycythemia or need for phototherapy (16). However, UCM has not been studied yet in infants with IUGR or placental insufficiency. Hence, International Liaison Committee on Resuscitation guidelines acknowledged that the ideal strategy of umbilical cord management in situations of fetal growth restriction and affected uteroplacental perfusion is not established yet with less provided guidance (8).

We conducted a case-control study to assess safety, feasibility, and impact of UCM on peripheral blood hematopoietic stem cells, hematological indices, and clinical outcomes in preterm with ante-natal evidence of placental insufficiency.

## Subjects and Methods

### Design and Setting

This was a case-control study. We compared hematological and clinical outcomes between prospectively recruited preterm neonates with antenatal evidence of placental insufficiency that underwent umbilical cord milking and historical controls for whom immediate cord clamping was done. We prospectively enrolled eligible neonates at the Neonatal Intensive Care Unit (NICU) of Mansoura University Children's Hospital, Mansoura, Egypt, from April 2019 through March 2020. Historical control neonates in PI+ICC group had been formerly enrolled in our previously published DCC trial conducted at our unit between September 2017 and March 2019. Institutional Research Board of Mansoura Faculty of Medicine approved the study (reference number: MS/17.07.44). Informed written consent was obtained prior to delivery from parents once the risk of preterm labor was identified.

### Included Subjects

Preterm neonates delivered <34^0/7^ weeks' gestation were included. Placental insufficiency was identified by fulfilling the following criteria: (1) evidence of fetal growth restriction was diagnosed when abdominal circumference of the fetus was below the 10th percentile for gestational age (17); (2) umbilical artery pulsatility index above the 95th percentile on umbilical artery Doppler assessment with or without absent end-diastolic flow (18).

### Excluded Subjects

We excluded neonates with suspected major congenital malformations, chromosomal anomalies, hydrops fetalis, multiple gestations, vaginal bleeding due to placental abruption or placental tear, and infants who required immediate resuscitative maneuvers at birth as in whom resuscitation could not be deferred.

### Study Groups

In the PI+UCM group, enrolled infants with placental insufficiency underwent UCM (infants were placed below the level of placenta, the umbilical cord was grasped at 20–25 cm distance from the infant and stripped gently toward the umbilicus for three times at a speed of ≈10 cm/s). Neonates in PI+ICC group had their umbilical cords immediately clamped within 10 s after delivery of the whole body of the infant at 2–3 cm from the umbilical stump without stripping. A third group of 30 preterm infants with normal feto-placental circulation managed with UCM was recruited.

### Patients Care Pathway

For measurement of CD34 percentage, 1 ml peripheral blood sample had been drawn within 60 min after delivery from all enrolled infants. CD34 percentage was determined using the three-color flow cytometry by conjugated monoclonal antibodies CD34 (phycoerythrin-texas red ECD, Beckman Coulter Inc., Danaher Corporation company, Pasadena, CA, USA). The laboratory technicians were not aware of the study groups' allocation. Samples for complete blood count (LH 450 counter, Beckman Coulter Inc., Danaher Corporation Company, Pasadena, CA, USA) were obtained in all infants within the first 60 min of life and at 2 months of age. All enrolled neonates received a uniform standard intensive supportive care as regards fluid management, phototherapy, inotropic support, respiratory support, and transfusion thresholds as per our unit policy.

### Outcomes

Our primary outcome was peripheral blood percentage of CD34 as an estimate of stem cell level. Secondary outcomes included CBC elements within the first hour of life; hemoglobin at 2 months of age as a marker for late-onset anemia; incidence of initial polycythemia (hematocrit level ≥ 65%); frequency of PRBCs transfusion during NICU stay; peak serum bilirubin during NICU stay; incidence of phototherapy, admission rectal temperature; hypothermia <36.5°C in the first 24 h of life; hypoglycemia in the first 24 h of life (defined as blood glucose level <47 mg/dl); episodes of hypotension in the first 24 h of life (defined as mean arterial blood pressure below the 10th percentile for age); boluses administered for management of hypotension; need for inotrope support during the first 24 h of age; duration of oxygen therapy; bronchopulmonary dysplasia (19); severe intra-ventricular hemorrhage (grade III or IV); necrotizing enterocolitis according to modified Bell staging criteria (20); culture-proven late-onset sepsis; length of hospital stay; and in-hospital mortality.

### Sample Size Calculation

Being a feasibility case-control study, we adopted a convenience sampling strategy assigning 30 infants in each arm. Based on our earlier research, approximately 26% of preterm infants priori allocated to placental transfusion intervention had been declined as they needed immediate resuscitative effort (16). Accordingly, we presumed that we should enroll 40 patients to PI+UCM group to attain our target sample size of 30.

### Statistical Analyses

We did two statistical comparisons. In the first, we compared outcomes between the two main groups: PI+UCM and PI+ICC. In the second, outcomes were compared between PI+UCM and No PI+UCM groups to validate significance of UCM maneuver in preterm infants with placental insufficiency. Statistical analysis was implemented using SPSS statistical software package (version 22; SPSS, Chicago, Illinois). Kolmogorov–Smirnov test was applied to assess the distribution of continuous data. Statistical comparison of nominal and continuous variables was done using Chi-square test or Fisher's exact test and Student *t*-test or Mann–Whitney test, respectively. Multiple linear regression and binary logistic regression were used to assess the relationship between umbilical cord management strategy and clinical outcomes among preterm neonates with placental insufficiency after adjustment for gestational age as a confounder. The strength and the direction of association between CD34 percentage and hemoglobin levels at 2 months of age were assessed using Spearman rank-order correlation coefficient test. The statistical significance cut-off level was set at *p* < 0.05. Data are expressed as mean ± standard deviation, median (interquartile range), or number (percentage) unless otherwise stated.

## Results

Our research team obtained informed consents from 36 mothers with placental insufficiency prior to delivery to be enrolled in PI+UCM group, of whom six were excluded from the analysis as infants required immediate resuscitative interventions according to attending physician's decision. The demographic data and clinical characteristics were matched in the two main comparison arms (PI+UCM and PI+ICC). Neonates in the PI+UCM arm had significantly lower birth weights with higher rates of birth weights <10th percentile for gestational age, higher rates of maternal diseases (specifically maternal hypertension), higher rates of antenatal prescription of magnesium sulfate and steroid, and higher percentages of cesarean section delivery compared with No PI+UCM group (Table 1).

**Table 1:** Demographic, prenatal, and baseline clinical characteristics.

**Variables**	**PI+UCM** **(Group A)** **(*n* = 30)**	**PI+ICC** **(Group B)** **(*n* = 30)**	**No PI+UCM** **(Group C)** **(*n* = 30)**
Maternal age (years)	25.66 ± 4.19	23.76 ± 3.56	27.73 ± 4.17
Maternal hemoglobin	12.18 ± 0.82	12.38 ± 0.77	12.38 ± 0.89
Maternal disease
•Diabetes	8 (26.66%)	3 (10%)	3 (10%)
•Hypertension	15 (50%)*	21 (70%)	3 (10%)
•Renal disease	2 (6.66%)	3 (10%)	1 (3.33%)
•Immune disease	1 (3.33%)	2 (6.67%)	4 (13.34%)
•Others	3 (10%)	1 (3.33%)	2 (6.66%)
Maternal UTI	5 (16.66%)	5 (16.66%)	5 (16.66%)
Chorioamnionitis	2 (6.66%)	5 (16.66%)	2 (6.66%)
Premature rupture of membranes	4 (13.34%)	5 (16.66%)	3 (10%)
Antenatal steroid	28 (93.33%)*	26 (86.66%)	19 (63.33%)
Antenatal magnesium sulfate	23 (76.66%)*	22 (73.33%)	9 (30%)
Post-partum hemorrhage	0	0	0
Gestational age (weeks)	31.10 ± 2.51	30.37 ± 1.21	31.97 ± 1.79
Cesarean section	27 (90%)*	28 (93.33%)	16 (53.33%)
Male	16 (53.33%)	16 (53.33%)	18 (60%)
Birth weight (grams)	978.33 ± 202.43*	893.33 ± 148.40	1629.83 ± 217.95
Small for gestational age	28 (93.33%)*	29 (96.66%)	3 (10%)
1-min APGAR score	5 (5–6)	5 (3–8)	5 (5–6)
5-min APGAR score	8 (7–10)	8 (6–9)	9 (7–9)
Meconium staining liquor	0 (0%)	0 (0%)	1 (3.33%)
Surfactant administration
•Single dose	3 (10%)	10 (33.33%)	8 (26.66%)
•More than one dose	9 (30%)	4 (13.43%)	2 (6.66%)

*Data are expressed as mean ± SD, median (interquartile range), or number (%)*.

*PI+UCM, placental insufficiency and umbilical cord milking; PI+ICC, placental insufficiency and immediate cord clamping; No PI+UCM, no placental insufficiency and umbilical cord milking*.

* *p < 0.05 compared to No PI+UCM*.

# *p < 0.05 compared to PI+ICC*.

**Figure 1:**
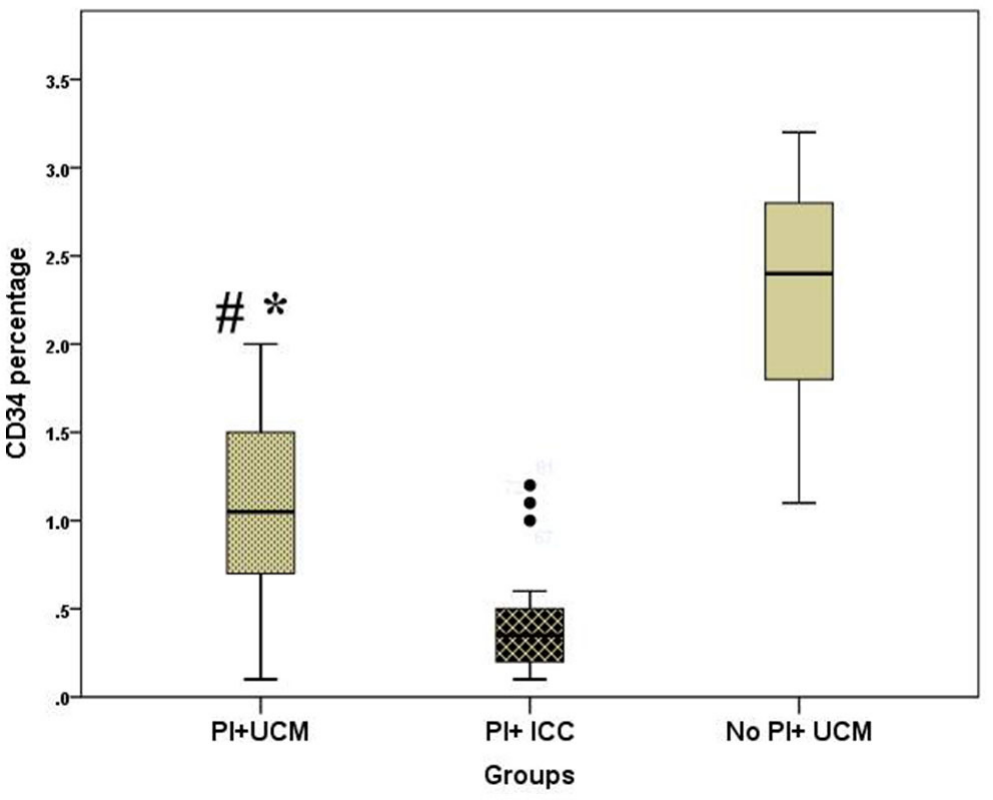
Box and whisker plot demonstrating peripheral blood CD34 percentage of the studied groups; PI+UCM (placental insufficiency and umbilical cord milking), PI+ICC (placental insufficiency and immediate cord clamping, No PI+UCM (no placental insufficiency and umbilical cord milking), *p < 0.05 compared to No PI+UCM, ^#^*p* < 0.05 compared to PI+ICC.

Peripheral blood CD34 percentage was significantly higher in the PI+UCM group compared with that in PI+ICC group [median (IQR) of 1.05 (0.70–1.50) vs. 0.35 (0.2–0.5), *p* < 0.0001]. Yet, neonates with placental insufficiency who underwent UCM still had significantly lower CD34 percentage compared to those in No PI+UCM group [median (IQR) of 1.05 (0.70–1.50) vs. 2.40 (1.80–2.80), *p* < 0.0001] (Figure 1).

Neonates in PI+UCM group had greater hemoglobin and hematocrit percentage within 24 h of life compared with those in PI+ICC group (Table 2). Among the whole cohort, only two neonates had a hematocrit >65 in PI+UCM group, but none of them were symptomatic for polycythemia-hyperviscosity syndrome. At 2 months of age, hemoglobin levels were significantly higher in neonates of PI+UCM group when compared to PI+ICC group (mean ± SD of 10.38 ± 1.40 vs. 9.53 ± 1.03, *p* = 0.013). Frequency of PRBCs transfusion was not significantly different between the studied groups. Neonates in PI+UCM group had higher peak TSB compared to those in PI+ICC. More patients in PI+UCM group required phototherapy compared to the PI+ICC (86 vs. 60%, *p* < 0.02) (Table 2).

**Table 2:** Hematologic parameters and outcomes between both groups.

**Variables**	**PI+UCM** **(Group A)** **(*n* = 30)**	**PI+ICC** **(Group B)** **(*n* = 30)**	**No PI+UCM** **(Group C)** **(*n* = 30)**
CD34 %	1.05 (0.70–1.500)^#*^	0.35 (0.2–0.5)	2.40 (1.80–2.80)
Admission hemoglobin (g/dl)	16.22 ± 1.69^#^	13.22 ± 1.24	16.51 ± 1.98
Admission hematocrit %	47.52 ± 7.26^#^	40.43 ± 2.49	49.97 ± 7.83
Admission WBCs (10^3^ cells/ml)	12.76 ± 4.98	17.77 ± 7.55	17.27 ± 6.05
Admission platelets (10^3^ cells/ml)	222.43 ± 68.53	235.10 ± 87.51	245.90 ± 68.41
Polycythemia (hematocrit ≥65%)	2 (6.66%)	0	0
Two month hemoglobin (g/dl)	10.38 ± 1.40^#^	9.53 ± 1.03^#^	10.53 ± 1.24
Frequency of PRBCs transfusion during NICU stay
•None	17 (56.66%)	10 (33.33%)	23 (76.66%)
•1	5 (16.66%)	11 (36.66%)	3 (10%)
•2	6 (20%)	1 (3.33%)	2 (6.66%)
•3	1 (3.33%)	2 (6.66%)	2 (6.66%)
•>3	1 (3.33%)	6 (20%)	0
Peak TSB (mg/dl)	11.27 ± 2.77^#^	6.97 ± 4.40	12.09 ± 2.15
Phototherapy need	26 (86.66%)^#^	18 (60%)	23 (76.66%)

*Data are expressed as mean ± SD, median (interquartile range), or number (%)*.

*PI+UCM, placental insufficiency and umbilical cord milking; PI+No UCM, placental insufficiency and immediate cord clamping; No PI+UCM, no placental insufficiency and umbilical cord milking*.

* *p < 0.05 compared to No PI+UCM*.

# *p < 0.05 compared to PI+ICC*.

Umbilical cord stripping was associated with a significant reduction in the duration of oxygen therapy compared to ICC in neonates with placental insufficiency. No statistically significant difference was noted between the studied groups as regards culture-proven sepsis, NEC, severe IVH, BPD, length of hospital stay, or in-hospital mortality (Table 3). Implementing multiple linear and binary logistic regression analyses revealed that UCM in preterm neonates with antenatal evidence of placental insufficiency resulted in greater CD34 percentage, higher initial hemoglobin level, higher peak serum bilirubin, significant increase of phototherapy initiation, and higher hemoglobin level at 2 months of age independent of the gestational age compared with ICC (Table 4). There was a positively moderate correlation between peripheral blood CD34 percentage and hemoglobin level at 2 months of age in preterm infants of the PI+UCM group (*r* = 0.44, *p* = 0.001).

**Table 3:** NICU course between groups.

**Variables**	**PI+UCM** **(Group A)** **(*n* = 30)**	**PI+ICC** **(Group B)** **(*n* = 30)**	**No PI+UCM** **(Group C)** **(*n* = 30)**
Admission temperature (°C)	36.56 ± 0.45	36.76 ± 0.44	36.83 ± 0.33
Hypothermic episodes within the 1st day of life			
•None	17 (56.66%)*	17 (56.66%)	26 (86.66%)
•Single	8 (26.66%)	6 (20.00%)	3 (10.00%)
•≥2	5 (16.66%)	7 (23.33%)	1 (3.33%)
Hypoglycemic episodes within the 1st day of life
•None	21 (70.00%)	17 (56.66%)	26 (86.66%)
•Single	6 (20.00%)	7 (23.33%)	2 (6.66%)
•≥2	3 (10.00%)	6 (20.00%)	2 (6.66%)
Hypotensive episodes within the 1st day of life
•None	21 (70.00%)	16 (53.33%)	25 (83.33%)
•Single	6 (20.00%)	6 (20.00%)	3 (10.00%)
•≥2	3 (10.00%)	8 (26.66%)	2 (6.66%)
Intravenous volume expander therapy within the 1st day of life	4 (13.33%)	5 (16.66%)	3 (10.00%)
Inotropic support within the 1st day of life	2 (6.66%)	3 (10.00%)	1 (3.33%)
Duration of oxygen therapy days	14.50 (6.50–25.00)^#^	22.5 (15.75–29.75)	17.50 (7.75–36.50)
Culture proven sepsis	4 (13.33%)	8 (26.66%)	7 (23.33%)
Necrotizing enterocolitis	1 (3.33%)	2 (6.66%)	1 (3.33%)
Severe intraventricular hemorrhage (Grade III or IV)	2 (6.66%)	2 (6.66%)	1 (3.33%)
Bronchopulmonary dysplasia	2 (6.66%)	4 (13.33%)	1 (3.33%)
Length of hospital stay (days)	32.50 (24.75–43.00)	25.00 (22.00–33.75)	25.00 (16.75–50.00)
In-hospital mortality	1 (3.33%)	3 (10.00%)	2 (6.66%)

*Data are expressed as mean ± SD, median (interquartile range), or number (%)*.

*PI+UCM, placental insufficiency and umbilical cord milking; PI+No UCM, placental insufficiency and immediate cord clamping; No PI+UCM, no placental insufficiency and umbilical cord milking*.

* *p < 0.05 compared to No PI+UCM*.

# *p < 0.05 compared to PI+ICC*.

**Table 4:** Multiple linear and binary logistic regression analysis of umbilical cord milking vs. immediate cord clamping regarding outcomes in preterm infants with ante-natal evidence of placental insufficiency.

**Characteristics**	**β coefficient**	**Odds ratio^**#**^** **(95% CI)**	***P*** **-value**
CD34 %	0.59		<0.001
Admission hemoglobin (g/dl)	3.06		<0.001
Two month hemoglobin (g/dl)	0.73		0.03
Peak TSB (mg/dl)	4.52		<0.001
Phototherapy need		28.18 (2.72–291.9)	0.005
Culture-proven sepsis		0.38 (0.10–1.43)	0.15
Need for PRBCs		0.49 (0.16–1.56)	0.23
Length of hospital stay (days)	5.33		0.13
Duration of oxygen therapy (days)	6.00		0.14
Necrotizing enterocolitis		0.21 (0.01–3.31)	0.26
BPD		0.38 (0.05–2.86)	0.35
Mortality		0.06 (0.002–2.35)	0.14
Combined outcome of death and/or BPD and/or severe IVH		0.35 (0.07–1.66)	0.18

# *Odds ratio adjusted for gestational age*.

## Discussion

A mounting evidence over the last decade noted that DCC at birth is more favorable compared to ICC in relatively stable preterm infants as evidenced by less rates of IVH, PRBCs transfusion, NEC, and death (1). UCM has been probed as an emerging alternative to DCC which provides placental transfusion benefits without deferring resuscitative efforts in preterm infants requiring immediate resuscitation at birth and those experiencing *in-utero* placental insufficiency. A recent meta-analysis found that UCM at birth in preterm infant was associated with lower incidence of IVH and need for PRBCs transfusion compared with ICC. On the other hand, UCM when compared to DCC was associated with increased IVH risk, particularly in extreme preterm neonates (2). The majority of currently available evidence has been pooled out from data of studies included preterm neonates born to mothers with intact placental circulation. Therefore, the best umbilical cord management strategy at birth in preterm infants with antenatal evidence of placental insufficiency is still uncertain.

We found that UCM upsurges peripheral blood hematopoietic progenitor stem cells (CD 34) alongside hemoglobin levels at birth and at 2 months postnatal age in premature neonates agonized placental insufficiency while *in utero* compared with ICC. On the other hand, among infants that underwent UCM at birth, peripheral CD34 was still significantly higher in neonates with normal feto-placental circulation compared to those with placental insufficiency with no significant difference regarding early and late hemoglobin levels. Furthermore, our results revealed no significant difference between studied groups regarding polycythemia. Similarly, Digal and colleagues observed higher venous hematocrit at 24 h of age and higher serum ferritin levels at 3 months of age in IUGR neonates born ≥28 weeks' gestational age managed by deferred cord clamping for 60 s vs. ICC (21). We previously reported that DCC for 60 s in infants with IUGR was associated with greater hemoglobin levels at 2 months of age without higher incidence of polycythemia (16). Furthermore, results of a recent network meta-analysis in non-IUGR preterm neonates showed higher peak hemoglobin within 24 h of age by 1.18 g/dl (95% CI: 0.65–1.71) in UCM group compared to ICC (3). In contrast, Wang et al. (15), in their subgroup analysis of preterm IUGR neonates, noted no difference in the initial hematocrit levels between deferring cord clamping for 30–45 s and ICC which may be attributed to shorter cord clamping time.

Preterm cord blood contained a greater concentration of circulating CD34+ hematopoietic stem cells and progenitors with a higher proliferative capacity than term cord blood (22). Katheria et al. (6) found that umbilical cord samples collected following UCM contained significantly lower percentage and absolute numbers of hematopoietic stem cells compared to DCC. However, post-milking residual umbilical cord blood revealed a higher ability to rescue irradiated bone marrow–depleted mice compared to post-deferred cord clamping residuals. The authors explained that phenomenon by the mechanical effect of milking which dislodged more mesenchyme stem cells from the stroma into cord blood which leads to more effective hematopoiesis. The aforementioned results supported our assumption that greater hemoglobin levels at 2 months postnatal age in infants with placental insufficiency managed by cord stripping compared to ICC may be due to ongoing hematopoiesis that served by additional amount stem cells delivered to neonates by cord milking.

We found that no significant difference between the two main groups (PI+UCM and PI+ICC) regarding need for PRBC transfusion during NICU stay. A recent pooled analysis of 9 trials included 688 preterm infants showing that UCM was associated with lower incidence of PRBC transfusion need compared to ICC (47.3 vs. 32.3%; 95% confidence interval, 0.23–0.53) (2). However, our results should be taken cautiously as PRBC transfusion requirements in preterm neonates may be affected by other comorbidities related to prematurity rather than umbilical cord management strategy such as sepsis, hemodynamic instability, and NEC. In addition, micro-sampling techniques for arterial blood gases and different laboratory testing are not available in our unit. A recent study reported that preterm neonates lose ~58% of their initial blood volume during first 14 days of NICU admission due to frequent sampling (23).

Infants in PI+UCM group showed higher peak TSB levels and more need of phototherapy compared to PI+ICC group. However, none of infants in our cohort required exchange transfusion. UCM is a well-tolerated umbilical cord strategy in infants born to mothers with placental insufficiency with no significant difference regarding admission rectal temperature, incidence of hypothermic and hypoglycemic episodes, and occurrence of early hypotension that necessitated volume expander and/or inotropic support compared to ICC. Furthermore, we did not observe any difference between both groups regarding length of hospital stay, in-hospital mortality, or major morbidities including severe IVH, NEC, BPD, and culture- proven late-onset sepsis. Similarly, Wang and colleague in a secondary analysis of IUGR infants found no difference between DCC and ICC as regards hospital course, mortality, and major morbidities. Infants in the milked group with placental insufficiency required shorter duration of oxygen therapy than ICC group. However, after adjustment for gestational age, results showed no significant difference between both groups.

The main concern as regards UCM implementation in preterm infants is IVH occurrence. This concern raised by a recent large trial halted early as the first interim subgroup analysis of 128 infants born <28 weeks' gestation suggested an increased risk of severe IVH with cord milking when compared to DCC (22 vs. 6%; *p* = 0.002). The IVH risk was not consistently reported among the whole cohort (474 infants) or among the subset of infants born at 28–32 weeks' gestation (24). Pratesi et al. explained lower IVH rates among infants underwent deferred cord clamping for at least 60 s by initiation of breathing in response to drying and gentle stimulation offered to babies before DCC which allows more physiological approach rather than isolated DCC (25). Nevertheless, their results should be interpreted cautiously as they did not report IVH occurrence rates in excluded infants who were immediately clamped at birth which might be higher than milked infants, alongside, premature termination of enrollment with diminished sample size. Moreover, in a recent pooled analysis of 10 trials that included 645 preterm infants, UCM compared with ICC was associated with significant decrease of IVH incidence with no difference regarding mortality between groups (2).

To our knowledge, this is the first study to evaluate the impact of UCM in infants born to mothers with placental insufficiency. We endorse the limitations of our study being a case control study with historical controls prone to selection bias and a relatively small sample size. Accordingly, a future randomized trial with appropriately powered sample size is justified to examine safety and benefits of UCM maneuver.

In conclusion, UCM in infants born to mothers with placental insufficiency is a safe and beneficial umbilical cord management strategy at birth. UCM resulted in greater CD34 percentage, higher initial hemoglobin level, and higher hemoglobin level at 2 months. However, cord milking was associated with higher peak TSB and need for phototherapy without higher incidence of polycythemia or exchange transfusion.

## Data Availability Statement

The raw data supporting the conclusions of this article will be made available on request to the corresponding author, without undue reservation.

## Ethics Statement

The studies involving human participants were reviewed and approved by Institutional Research Board of Mansoura Faculty of Medicine. Written informed consent to participate in this study was provided by the participants' legal guardian/next of kin.

## Author Contributions

NN and IN formulated the hypothesis, designed the study protocol, supervised recruitment of participants, drafted the initial manuscript, and adapted it according to other authors' reviews. IN designed the statistical analysis and analyzed the data. MS and HA helped in designing the study, supervised data collection, analyzed the results, and critically appraised the manuscript. MN recruited patients, collected the data, and approved the final manuscript. MD implemented laboratory work and reviewed the manuscript. AG contributed to study design, supervising umbilical cord management maneuvers at birth and obstetric data collection, and reviewed the final manuscript. All authors approved the final manuscript as submitted and agreed to be accountable for all aspects of the work.

## Conflict of Interest

The authors declare that the research was conducted in the absence of any commercial or financial relationships that could be construed as a potential conflict of interest.

## Publisher's Note

All claims expressed in this article are solely those of the authors and do not necessarily represent those of their affiliated organizations, or those of the publisher, the editors and the reviewers. Any product that may be evaluated in this article, or claim that may be made by its manufacturer, is not guaranteed or endorsed by the publisher.

## Abbreviations

BPD: Bronchopulmonary dysplasia
CD34: Cluster of differentiation 34
CPAP: Continuous positive airway pressure
DCC: Deferred cord clamping
ELBW: Extreme low birth weight infant
ICC: Immediate cord clamping
IUGR: Intrauterine growth retardation
IVH: Intraventricular hemorrhage
NEC: Necrotizing enterocolitis
NICU: Neonatal intensive care unit
NO PI+UCM: No placental insufficiency and umbilical cord milking
PI+ICC: placental insufficiency and immediate cord clamping
PI+UCM: placental insufficiency and umbilical cord milking
PRBCs: Packed Red blood cells
SGA: Small for gestational age
TSB: Total serum bilirubin
UCM: Umbilical Cord Milking.
